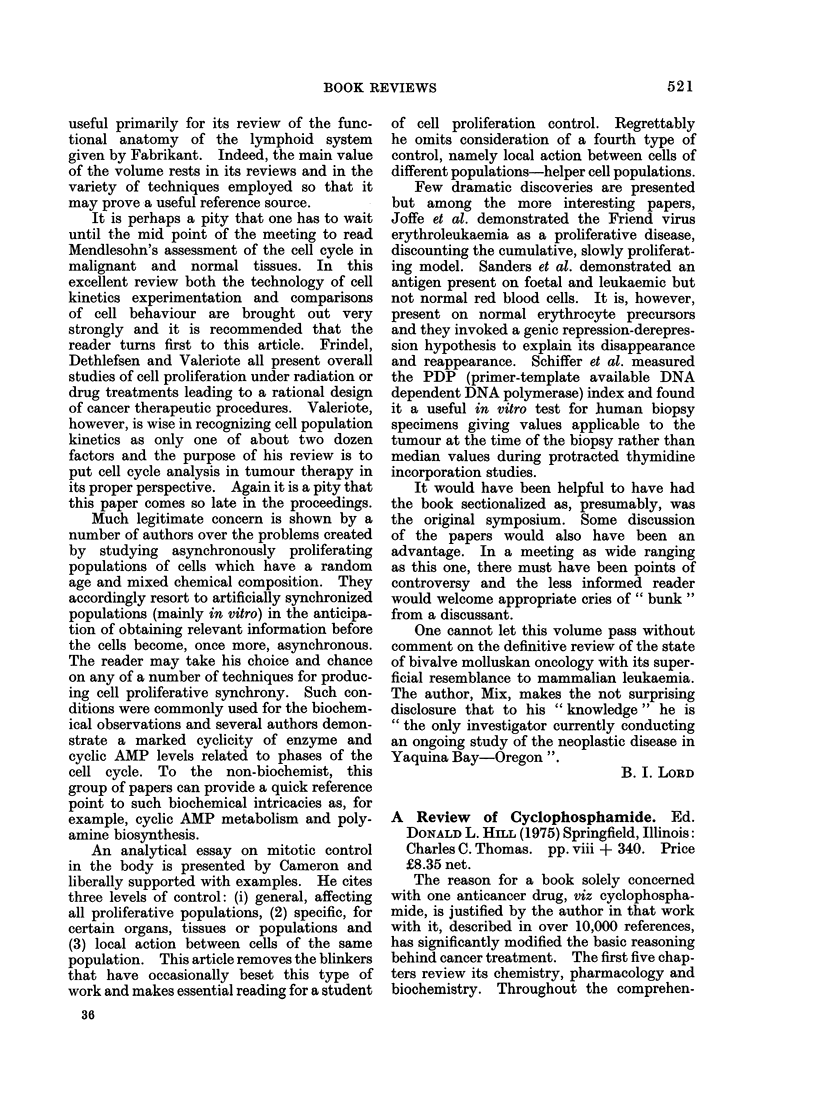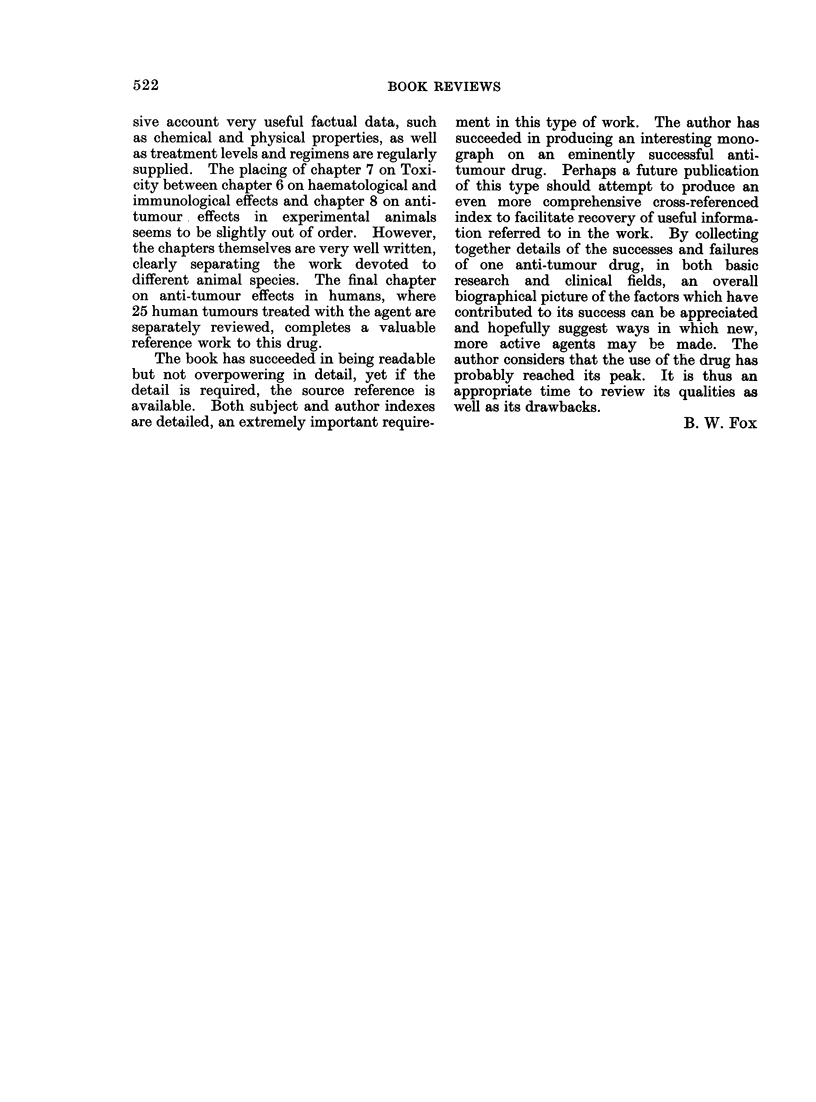# A Review of Cyclophosphamide

**Published:** 1975-10

**Authors:** B. W. Fox


**A Review of Cyclophosphamide.** Ed. Donald L. Hill (1975) Springfield, Illinois: Charles C. Thomas. pp. viii + 340. Price £8.35 net.

The reason for a book solely concerned with one anticancer drug, *viz* cyclophosphamide, is justified by the author in that work with it, described in over 10,000 references, has significantly modified the basic reasoning behind cancer treatment. The first five chapters review its chemistry, pharmacology and biochemistry. Throughout the comprehensive account very useful factual data, such as chemical and physical properties, as well as treatment levels and regimens are regularly supplied. The placing of chapter 7 on Toxicity between chapter 6 on haematological and immunological effects and chapter 8 on anti-tumour effects in experimental animals seems to be slightly out of order. However, the chapters themselves are very well written, clearly separating the work devoted to different animal species. The final chapter on anti-tumour effects in humans, where 25 human tumours treated with the agent are separately reviewed, completes a valuable reference work to this drug.

The book has succeeded in being readable but not overpowering in detail, yet if the detail is required, the source reference is available. Both subject and author indexes are detailed, an extremely important requirement in this type of work. The author has succeeded in producing an interesting monograph on an eminently successful anti-tumour drug. Perhaps a future publication of this type should attempt to produce an even more comprehensive cross-referenced index to facilitate recovery of useful information referred to in the work. By collecting together details of the successes and failures of one anti-tumour drug, in both basic research and clinical fields, an overall biographical picture of the factors which have contributed to its success can be appreciated and hopefully suggest ways in which new, more active agents may be made. The author considers that the use of the drug has probably reached its peak. It is thus an appropriate time to review its qualities as well as its drawbacks.

B. W. Fox